# Human papillomavirus infection in women undergoing in-vitro fertilization: effects on embryo development kinetics and live birth rate

**DOI:** 10.1186/s12958-023-01091-9

**Published:** 2023-04-24

**Authors:** Federica Zullo, Valentina Fiano, Anna Gillio-Tos, Sara Leoncini, Ginevra Nesi, Luigia Macrì, Mario Preti, Alessandro Rolfo, Chiara Benedetto, Alberto Revelli, Laura De Marco

**Affiliations:** 1 Gynecology and Obstetrics 1U, Physiopathology of Reproduction and IVF Unit, Dept. of SurgicalSciences, Sant’Anna Hospital, University of Torino, Torino, Italy; 2 Centre for Cervical Cancer Screening, City of Health and Science Hospital, Turin, Italy; 3 Gynecology and Obstetrics 2U, Physiopathology of Reproduction and IVF Unit, Dept. of Surgical Sciences, Sant’Anna Hospital, University of Torino, Via Ventimiglia 3, Torino, 10126 Italy; 4 Cancer Epidemiology Unit, Department of Medical Sciences, University of Turin and CPO Piemonte, Turin, Italy

**Keywords:** Human papilloma virus, Endometriosis, IVF outcome, Embryonic development, Time-lapse, Live birth rate

## Abstract

**Backgroud:**

Several studies showed that human papillomavirus (HPV) affects male fertility, but its impact on female fertility and in vitro fertilization (IVF) outcome is not yet clear.

**Methods:**

Objective of this observational, prospective, cohort study was to evaluate the prevalence of HPV infection in women candidate to IVF, and the effects of HPV infection on the kinetic of embryonic development and on IVF outcome. A total number of 457 women candidate to IVF were submitted to HR-HPV test; among them, 326 underwent their first IVF cycle and were included in the analysis on IVF results.

**Results:**

8.9% of women candidate to IVF were HPV-positive, HPV16 being the most prevalent genotype. Among the infertility causes, endometriosis was significantly more frequent in HPV-positive than in negative women (31.6% vs. 10.1%; p < 0.01). Granulosa and endometrial cells resulted HPV-positive in 61% and 48% of the women having HPV-positive cervical swab, respectively. Comparing HPV-positive and negative women at their first IVF cycle, no significant difference was observed in the responsiveness to controlled ovarian stimulation (COS) in terms of number and maturity of retrieved oocytes, and of fertilization rate. The mean morphological embryo score was comparable in the two groups; embryos of HPV-positive women showed a quicker development in the early stages, with a significantly shorter interval between the appearance of pronuclei and their fusion. In the following days, embryo kinetic was comparable in the two groups until the early blastocyst stage, when embryos of HPV-positive women became significantly slower than those of HPV-negative women. Overall, these differences did not affect live birth rate/started cycle, that was comparable in HPV-positive and negative women (22.2 and 28.1%, respectively).

**Conclusions:**

(a) the prevalence of HPV infection in women candidate to IVF is similar to that observed in the general female population of the same age range; (b) HPV infection migrates along the female genital apparatus, involving also the endometrium and the ovary, and perhaps participates in the genesis of pelvic endometriosis; (c) HPV slightly affects the developmental kinetic of in vitro-produced embryos, but does not exert an effect on live birth rate.

## Introduction

Human papillomavirus (HPV) infection represents a quite frequent sexually transmitted disease, as half of the sexually active males and females may be affected during lifetime [1]. The main risk factors for HPV infection are low socio-economic status, geographic location, lifestyle, high number of sexual partners, and age. A higher risk of HPV infection is documented in women below 25 years and between 55 and 64 years [2, 3].

HPV is a DNA-virus with specific tropism for human epithelial tissue. There are almost 200 HPV different genotypes, classified as low risk (LR-HPV) or high risk (HR-HPV) on the basis of their ability to induce neoplastic transformation of the infected cells [4, 5]. Although HPV infection is often transient and asymptomatic, HR-HPVs represent the main cause of genital lesion with progression to cancer. Cervical cancer, in turn, is the fourth most common female cancer worldwide (the second in low socio-economic index areas) [6]; moreover, HR-HPV infection is also associated with cancers of the vagina, vulva, anus, penis, oropharynx and oral cavity [7, 8].

Recent studies suggest that HPV may affect fertility and perhaps also the efficacy of in-vitro fertilization (IVF) [9–11]. In the male, HPV effects on semen are rather well known: reduction of sperm motility [12–14], and increase of sperm DNA fragmentation [15, 16]. HPV can even bind spermatozoa along the equatorial region of the head, likely transmitting viral DNA to oocytes, and finally influencing embryonic development [17]. In the mouse model, in fact, sperm HPV infection was shown to decrease the blastocyst formation rate, inhibit the hatching process and reduce the embryo chance to implant [18, 19].

The effect(s) of HPV on female fertility are unclear. It was reported that HPV-related cervical cytology abnormalities are more common in infertile women than in the general population [20]. Further, a correlation between HPV and pelvic endometriosis, a known cause of infertility, was observed [21]. Some studies suggest that female HPV infection could even lower the success rate of in vitro fertilization (IVF) [22, 23], increasing the risk of miscarriage, and finally decreasing the live birth rate [24–26]. These findings, however, were not confirmed by other reports [27–30], and the topic is still matter of discussion.

The present study was designed as an observational prospective cohort study, and was aimed at evaluating both the prevalence of HPV in women undergoing IVF, and the impact of HPV infection on embryo morphokinetic and IVF outcome.

## Materials and methods

### Study population

A total number of 457 women with couple’s infertility, aged 30–43 years, in the waiting list of IVF program at the Physiopathology of Reproduction and IVF Unit of S. Anna Hospital (University of Turin), were submitted to a cervical swab for HR-HPV test. Among them, 326 underwent their first IVF treatment in the study time period (January – October 2021) and were included in the analysis of IVF outcome. For those who resulted positive for HPV in the cervical swab, HPV genotyping and cervical cytology (PAP test) were performed. Moreover, in case of cervical positivity, the presence of HPV-DNA was also tested in endometrial cells (collected during a mock embryo-transfer before starting IVF procedure) and in granulosa cells (collected from the follicular fluid at the time of oocyte retrieval).

The study was carried out in accordance to the Declaration of Helsinki and was authorized by the local Ethical Committee (Ref. number 0000055). Signed informed consent was obtained from all patients.

### Cervical cells sampling, HR-HPV test, PAP test, endometrial cells sampling

Cervical sampling was accomplished approximately one month before starting IVF procedure using a specific sampling set (ThinPrep, Hologic), gently scraping cells from the cervical canal and from the internal and external portion of the cervix. Cervical samples were stored in a 10 ml transport medium (PreservCyt® ,Hologic, ThinPrep), and labelled with an ID number.

Briefly, cervical samples were processed by QIAsymphony®DSP (Qiagen, Hilden, Germany), an automatic system based on magnetic particle technology, for the isolation of human cervical cells from the preserving solution. Subsequently, isolated cervical cells were tested by the Hybrid Capture 2 assay High-Risk HPV DNA test (HC2, Qiagen, Hilden, Germany) by using the Rapid Capture System® (RCS; Qiagen, Hilden, Germany) according to the manufacturer’s instructions. HC2 test is a nucleic acid hybridization assay with signal amplification for qualitative detection of 14 HPV types in cervical cells (HPV16, -18, -31, -33, -35, -18, -39, -45, -51, -52, -56, -58, -59, and − 68). HC2 results were expressed as the ratio between the specimen’s light emission (relative light units [RLU]) and the mean of three concurrently tested positive controls (CO). Samples were considered positive when the ratio (RLU/CO) was ≥ 1 [31].

In case of positivity to HPV test, a cytological smear was set up from the same sample and was stained using Papanicolaou staining (Pap Test) in order to detect morphological features of any eventual cervical lesion. Cytology was classified using the Bethesda 2001 system: data were classified as negative for intraepithelial lesions of malignancy (NILM), low grade (LSIL, ASC-US, and AGC), or high grade (ASC-H, HSIL, adenocarcinoma in situ-AIS-, and cancer).

Endometrial cell sampling was performed using a modified embryo transfer catheter consisting of two components: an outer guide and an inner soft part; first, the guide was inserted into the cervical canal, avoiding contact with the vaginal walls; the inner soft catheter was then gently inserted into the guide and advanced until reaching the endometrium inside the uterine cavity. After rotating the inner part tip in order to collect endometrial cells, it was retracted without exposition the vaginal environment, its distal end (approximately 5–10 mm) was cut using sterile scissors and immediately placed in sterile tubes for HPV infection assessment.

### DNA extraction, HPV-DNA detection and genotyping

DNA was extracted from cervical, endometrial and granulosa cells by using the QIAamp DNA Mini Kit® (Qiagen, Hilden, Germany), following the manufacturer’s instruction. Extracted DNA was quantified at the NanoDrop® ND-1000 spectrophotometer and, in order to check DNA quality, the β-globin (152 base pairs length (bp) housekeeping gene was amplified from the clinical samples using iCycleriQ™ Real-Time PCRDetection System (BioRad®, Hercules, CA, USA). PCR reaction contained 2X SYBR Green Supermix (BioRad®), 0.3 µM of β-globin primers [forward (5’GAAGAGCCAAGGACAGGTAC3’); reverse (5’CAACTTCATCCACGTTCACC3’)] [32], and 2 µl of DNA sample. Each sample was analysed in duplicate, and a control without DNA was included in each run.

To test HPV presence, a 150 bp L1 region was amplified with GP5+/GP6 + consensus primers, allowing detection of a broad range of HPV types [33, 34]. Consensus primers GP5+ (5’TTTGTTACTGTGGTAGATACTAC3’) and GP6+ (5’GAAAAATAAACTGTAAATCATATT3’) [35] were used. A positive (SIHA cell lines) and a negative control (without DNA) were added to each PCR run. The presence of PCR amplicon was checked on 2% agarose gel stained with ethidium bromide.

Samples testing positive for GP5+/GP6 + PCR were genotyped by using INNO-LiPA® HPV Genotyping Extra II Amplification test (FUJIREBIO, Belgium). This assay can identify 32 different HPV genotypes at the same time using reverse hybridization, after a preliminary step of amplification. PCR reactions were carried out in thermocycler (GeneAmp PCR System 9700) using SPF10 biotinylated consensus primers.

The biotinylated amplicons were denatured and hybridized with specific oligonucleotide probes fixed on membrane strips. After reverse hybridization, they were incubated with BCIP/NBT chromogen at 20–25° C on a shaker yielding a purple precipitate, and the results were visually interpreted (INNO-LiPA® HPV Genotyping Extra II Ampmanual).

### IVF procedure

Controlled Ovarian Stimulation (COS) with gonadotropins was accomplished according to standardized protocols. Follicular growth was monitored by serial transvaginal ultrasound (TV-US) and measurement of circulating estradiol (E2) every second day from day 7. When at least two follicles reached 18 mm mean diameter, with appropriate E2 levels, a single subcutaneous injection of 10.000 IU hCG (Gonasi HP, IBSA, Pambio Noranco, Switzerland) was administered to trigger ovulation.

Transvaginal US-guided oocyte pick-up (OPU) was performed 35–37 h later under local anesthesia (paracervical block). Follicular fluid was aspirated and immediately observed under stereomicroscope. Cumulus-oocytes complexes (COCs) were washed in buffered medium (Follicle Flush Buffer – COOK Medical, Ireland) and incubated in controlled atmosphere (37 °C, 5% O2, 6.5% CO2) using appropriate culture medium (Fertilization Medium - COOK Medical, Ireland).

The same day of OPU, semen samples were examined to assess sperm concentration, motility and morphology according to the World Health Organization guidelines (WHO laboratory manual, 2010); then they were prepared by density gradient centrifugation in order to select normally motile and morphologically normal spermatozoa. A few hours after OPU, oocytes were fertilized by conventional IVF or ICSI (Intra-Cytoplasmic Sperm Injection) according to semen parameters.

Normal fertilization was assessed 16–18 h later (Day 1) by evaluating the presence of two pronuclei (2PN) and the extrusion of the second polar body.

Embryos were cultured in pre-equilibrated cleavage medium (Cleavage Medium - COOK Medical, Ireland) overlain with mineral oil up to day 3 of development; at this stage, a change of medium was performed and a new one (Blastocyst Medium - COOK Medical, Ireland) was kept until the blastocyst stage (day 5–6). Embryo morphological evaluation was first performed on day 2 using the Integrated Morphology Cleavage Score (IMCS) [36], and then on day 5 according to The Istanbul Consensus Workshop [37]. In order to obtain information on the kinetic of embryonic development, embryos were cultured in micro-wells (one zygote/micro-well) using the Geri plus® time-lapse system (Genea Biomed, Germany) with integrated embryo monitoring system. Geri plus ® is a last generation incubator which allows to continuously (24 h/24) observe embryo growth at regular time intervals (every 5 min) without modifying culture conditions. All videos obtained by Geri plus® were analyzed, and the following morphokinetic parameters were considered: time of pronuclear appearance (tPNa), pronuclear fading (tPNf), completion of cleavage to two, three, four and eight cells (t2, t3, t4, and t8 respectively), blastulation start (tSB), full blastocyst development completion (tB), initiation of hatching process (tHN). The following time intervals were also calculated: tPNf-tPNa, t2-tPNf, t3-t2, t4-t3, t4-t2 and t8-t4 [38].

Embryo transfer (ET) in uterus was performed on day 5 using the soft catheter Sydney Guardia (COOK Medical, Ireland) under transvaginal US guidance, as previously reported by our group [39].

### Statistical analysis

The primary endpoint for power calculation was the prevalence of HPV infection among infertile women candidate to IVF. Accordingly, the power calculation was made as follows: considering the available epidemiologic data, we hypothesized that HPV infection could be 10% more frequent among women undergoing IVF than in the general population, and 450 subjects would have allowed to reach a statistical power of 80% with 0.05 alpha error.

Analyses were performed subdividing women undergoing IVF according to the presence or absence of HPV infection in the cervical swab. The data normal distribution was assessed using Kolmogorov-Smirnov test. Comparison between HPV-positive and -negative women was performed using the Student t-test (continuous variables) or the χ^2^ test (categorical variables), and the results were expressed by mean and standard deviation or by percentage. All statistical tests were for unpaired data and two-sided; a p-value < 0.05 was considered significant.

## Results

### Prevalence of HPV infection and genotyping

Among the 457 recruited women, 41 (8.9%) resulted HPV-positive at the cervical swab. Their mean age was 36.5 ± 3.7 years, and HPV prevalence was higher in younger women and decreased linearly with age, similarly to what observed in the general population of the same age range (not shown). Among infertility causes, endometriosis was the only to be significantly more frequent in HR-HPV-positive than in negative women (31.6% vs. 10.1%; p < 0.01).

In case of HPV-positivity, a Pap Test was performed to evaluate the presence of cytological lesions: 80% of patients resulted negative for intraepithelial lesions or malignancy (NILM), 16.6% and 3.3% had LSIL or HSIL, respectively. After genotyping, HPV16 was the most prevalent genotype (17.8%), followed by HPV18 and 59 (8.21%), 45 and 66 (6.85%), 31, 33, 51 and 52 (4.11%). According to what suggested by experts of the Low Genital Tract Infections Unit of our Institution, patients with HSIL were temporarily excluded from IVF program, whereas patients with LSIL were admitted.

Interestingly enough, granulosa and endometrial cells resulted positive for HPV in 61% (25/41) and 48% (20/41) of women who were HPV-positive at the cervical swab, respectively. Of note, endometrial biopsy was performed using a modified Vabra catheter having an outer part in order to avoid any contamination from the cervix.

### IVF outcome

Basal clinical characteristics and IVF outcome of the 326 patients who underwent their first IVF in the time period of the study are summarized in Tables 1 and 2, respectively. Comparing 27 HPV-positive and 299 HPV-negative women, no significant differences were observed for the main basal characteristics: age, BMI, AMH, basal (day 3) FSH and antral follicle count (AFC) (Table 1).

**Table 1:** Clinical characteristics of the 326 women (27 HPV-positive and 299 HPV-negative) undergoing their first IVF cycle

	HPV-positive (n = 27)	HPV-negative (n = 299)	p
*Age (years)*	36.4 ± 3.7	36.5 ± 3.6	0,855
*BMI (kg/m* ^*2*^ *)*	21.8 ± 3.3	22.5 ± 3.8	0,156
*AMH (ng/ml)*	3.4 ± 4.2	3.2 ± 3.4	0,869
*Day 3 FSH (IU/l)*	8.5 ± 2.1	8.0 ± 4.3	0,394
*Antral Follicle Count (AFC)*	12.7 ± 9.9	13.1 ± 7.3	0,830

Data are shown as mean ± SD or as percentage

The ovarian responsiveness to COS was comparable in the two groups, leading to similar oocyte yield; even egg competence appeared similar, with comparable fertilization rate, cleavage rate, and embryo availability (Table 2). The embryo morphological score was comparable in HPV-positive and negative women; all patients had at least one blastocyst to be transferred in uterus, and all received a single embryo transfer. A total number of 108 clinical (ultrasound-confirmed) pregnancies were observed (Clinical Pregnancy Rate/started cycle: 33.1%); miscarriage rate was not statistically different for HPV-positive woman (25% vs. 15%, p = 0.455). Finally, live birth rate/started cycle (LBR/SC) was comparable (22.2% for HPV-positive vs. 28.1% for HPV-negative women, p = 0.513) (Table 2).

**Table 2:** Clinical outcome of IVF treatment of 326 HPV-tested women (27 HPV-positive and 299 HPV-negative).

	HPV-positive (n = 27)	HPV-negative (n = 299)	p
*Total exogenous FSH (IU)*	2491 ± 878	2640 ± 998	0,409
Peak endometrial thickness (mm)	10.4 ± 2.0	10.2 ± 2.0	0,677
*Peak E2 (pg/ml)*	2200 ± 2198	1950 ± 1241	0,565
*Retrieved oocytes (n)*	8.5 ± 7.2	8.3 ± 5.8	0,890
*Mature (MII) oocytes (%)*	73.5 ± 29.4	82.2 ± 18.3	0,148
*Fertilized (2PN) oocytes (%)*	65.6 ± 28.3	71.3 ± 24.1	0,343
*Cleaved embryos (%)*	97.8 ± 10.4	96.4 ± 12.3	0,550
*Transferred embryos (n)*	33	449	
*Implantation rate (%)*	24.2	25.8	0,840
*Pregnancy rate/started cycle (%)*	33.3	35.8	0,799
*Clinical Pregnancy Rate/started cycle (%)*	29.6	33.4	0,687
*Ongoing Pregnancy Rate/started cycle(%)*	22.2	28.4	0,491
*Miscarriage rate (%)*	25	15	0,455
*Live Birth Rate/started cycle (%)*	22.2	28.1	0,513

Data are shown as mean ± SD or as percentage

Overall, 845 embryos were analyzed using the Time-Lapse System; 446 reached the blastocyst stage, among which 202 were obtained from HPV-positive and 244 from negative women (Table 3). In the early development stages, embryos in the HPV-positive group showed a quicker kinetic, with a significantly shorter pronuclei fading time (tPNf) (23.40 ± 2.64 vs. 24.95 ± 5.39 h, p = 0.005) and a significantly shorter interval between the appearance of pronuclei and their fading (tPNf-tPNa) (15.51 ± 2.83 vs. 17.40 ± 5.90 h, p = 0.014). In the following days, embryo kinetic was comparable between the two groups until the blastocyst stage (tB), when the embryos of HPV-positive women became significantly slower than those of HR-HPV-negative subjects (tB 122.76 ± 9.04 vs.118.90 ± 10.20 min, p = 0.049) (Table 3).

**Table 3:** Embryo morphokinetic parameters recorded by TLS GERI plus®

	Embryos from HPV-positive women (n = 202)	Embryos from HPV-negative women (n = 244)	Δ time	p
*tPNa (h)*	7.97 ± 2.07	7.47 ± 1.89	0.50	0,150
*tPNf (h)*	23.40 ± 2.64	24.95 ± 5.39	− 1.55	**0,005**
*t2 (h)*	27.00 ± 3.75	28.15 ± 4.87	− 1.15	0,051
*t3 (h)*	36.99 ± 4.90	37.91 ± 10.16	− 0.92	0,389
*t4 (h)*	39.03 ± 7.82	40.12 ± 9.42	− 1.09	0,369
*t8 (h)*	62.15 ± 13.08	62.19 ± 14.78	− 0.04	0,986
*tSB (h)*	102.32 ± 8.72	100.25 ± 9.58	2.07	0,205
*tB (h)*	122.76 ± 9.04	118.90 ± 10.20	3.86	**0,049**
*tHN (h)*	115.55 ± 7.92	113.29 ± 11.41	2.26	0,429
*tPNf – tPNa (h)*	15.51 ± 2.83	17.40 ± 5.90	− 1.89	**0,014**
*t2 – tPNf (h)*	3.58 ± 2.97	3.52 ± 2.26	0.06	0,874
*t3 – t2 (h)*	10.19 ± 3.98	9.72 ± 8.52	0.47	0,594
*t4 – t3 (h)*	2.42 ± 4.72	3.08 ± 5.49	− 0.66	0,367
*t4 – t2 (h)*	12.55 ± 6.65	12.30 ± 7.21	0.25	0,798
*t8 – t4 (h)*	23.34 ± 11.64	24.13 ± 13.27	− 0.79	0,690

Time and time intervals are shown for 202 embryos of HPV-positive women and 244 embryos of HPV-negative women. The ∆Time column shows the difference between embryos of HR-HPV-positive women and those of negative women for each morphokinetic parameter. Values are expressed as mean ± SD

The IVF outcome of patients with or without HPV infection in granulosa cells was comparable for all clinical parameters, including the rate of mature oocytes (69.3 ± 34% vs. 79.3 ± 21.8%, respectively, p = 0.37), that of fertilized oocytes (57.8 ± 33.6% vs. 74.8 ± 17.8%, respectively, p = 0.13), and the embryo morphological score (7.2 ± 1.6 vs. 6.5 ± 1.9, p = 0.37). Also the morphokinetic analysis of embryos belonging to patients with granulosa-positive or negative cells was not significantly different, as well as the LBR/SC (not shown).

Differently, HPV-positivity of endometrial cells resulted in a much lower LBR/SC (14.7%) than that observed in women with HPV-negative endometrium (42.8%), but the difference was not statistically significant, likely due to the limited number of observations.

## Discussion

The role of HPV in human infertility was previously investigated in a few studies. A higher incidence of HPV infection was demonstrated in the semen of infertile men [40–42], and the HPV-related reduction of sperm motility [12–14] and increased sperm DNA fragmentation [15, 16] was reported.

Differently, the effect(s) of HPV on the woman’s reproductive potential was never clearly defined, although some studies suggested that HPV might negatively affect woman’s fertility. It was reported, in fact, that HPV-related cervical cytology abnormalities were more common in infertile women than in the general population [20], and a correlation between HPV and pelvic endometriosis, a known cause of infertility, was observed [21]. Moreover, some studies reported that female HPV infection could lower the pregnancy rate in IVF [22, 23] and increase the risk of miscarriage, finally decreasing the live birth rate [24–26]. A possible mechanism underlying this effect could be the binding of HPV to the equatorial region of sperm head, with transmission of the virus DNA to the embryo [17]. In the mouse model, in fact, sperm HPV infection decreases the blastocyst formation rate, inhibits hatching process and reduces the embryo chance to implant [18, 19]. All claimed detrimental effects of HPV over human IVF outcome, however, were not confirmed by other studies [27–30, 43, 44].

In the present study, we observed a group of 457 women candidate to IVF for various indications: all underwent HPV testing on a cervical swab, and the prevalence of HPV infection was 8.9%, in line with the general female population of the same age range in our geographical area. A higher incidence of HPV infection was observed at younger age, and even this was common to both infertile women and the general female population. Different causes of infertility were similarly distributed in couples with HPV-positive or negative cervical swab, with the exception of endometriosis, that was significantly more frequent among HPV-positive women. As hypothesized by others [21], our molecular analyses confirmed that HPV is able to move along the female genital tract and infect also the endometrium and the ovary (granulosa cells) in a rather high proportion of women with cervical positivity. As HPV can reach the abdominal cavity, it could contribute to the establishment of pelvic endometriosis by enhancing pro-inflammatory pathways and weakening the local immune response against endometriosis cells. Endometriosis, however, is frequently observed also in adolescents and in non sexually active women: the association with HPV could simply reflect a higher vulnerability of women with endometriosis to the virus, possibly linked to defects in local immunity, or a lower capability to clear it. The hypothesis of an active involvement of HPV in the onset of endometriosis is worth new studies, including in vitro tests. Also the eventual oncogenic effect of HPV at the endometrial and ovarian level should be further investigated. Indeed the presence of HPV at the endometrial level suggests a possible role in the malignant transformation of the tissue.

As for the efficacy of IVF, we evaluated only first IVF cycles ending with blastocyst transfer in order to compare homogeneous groups of HPV-positive and negative women, excluding other factors potentially able to influence IVF outcome. Overall, our observations do not suggest any negative effect of female HPV infection on the key steps of IVF procedure: in fact, HPV-positive women had a similar responsiveness to COS, similar oocyte maturation rate, fertilization rate and embryo morphological quality than their negative controls. Finally, the clinical pregnancy rate and the miscarriage rate were comparable (although the latter was non-significantly higher in HPV-positive women), leading to a similar LBR/started cycle.

Using the time-lapse technology to detect any possible, even subtle, effect of HPV on embryonic development, we observed only small differences, not leading to a significantly different clinical outcome. Indeed embryos of HPV-positive women showed a quicker development in the early stages, but later on, in the late stages of growth to blastocyst, they became slower than those of HPV-negative women, in agreement to what observed in the mouse by Henneberg et al. [19], who reported a slower blastulation and delayed hatching of embryos exposed to HPV.

In patients with HPV in granulosa cells, the virus did not seem to affect follicular development, oocyte maturation and competence. Differently, the presence of HPV in the endometrium was apparently associated with a lower embryo implantation rate, but this observation was made on such a small number of cases, that it definitely needs to be eventually confirmed by novel studies.

## Conclusions

In conclusion, the present study (a) confirms that the prevalence of HPV infection in women candidate to IVF is comparable to the general female population of the same age range, (b) shows that HPV can move along the female genital apparatus and could be somehow involved in the genesis of pelvic endometriosis, and (c) shows that HPV slightly affects the developmental kinetic of in vitro-produced embryos, without significantly affecting the main IVF outcomes, including the live birth rate per started cycle.

## Data Availability

The subjects included in this study have not concomitantly been involved in other randomized trials. Data regarding any of the subjects in the study have not been previously published. Data will be made available to the editors of the journal for review or query upon request.

## Abbreviations

PRC: polymerase chain reaction
hCG: human chorionic gonadotropin
LSIL: low-grade squamous intraepithelial lesion
HSIL: high-grade squamous intraepithelial lesion
BMI: body mass index
AMH: anti-Mῢllerian hormone
FSH: follicle-stimulation hormone
LBR: live birth rate
